# Outcomes and factors associated with severe malaria-related anaemia in paediatric patients in northern Nigeria

**DOI:** 10.4314/gmj.v58i4.3

**Published:** 2024-12

**Authors:** Olayinka R Ibrahim, Amudalat Issa, Michael A Alao, Bello M Suleiman

**Affiliations:** 1 Department of Paediatrics and Child Health, University of Ilorin Teaching Hospital, Ilorin, Nigeria; 2 Department of Paediatrics, Children Specialist Hospital, Ilorin, Nigeria; 3 Department of Paediatrics, University of Ibadan & University College Hospital, Ibadan, Nigeria; 4 Department of Paediatrics, Federal Teaching Hospital, Katsina, Nigeria

**Keywords:** Anaemia, plasmodium falciparum, child, hospitalisation outcomes, Nigeria

## Abstract

**Objective:**

To determine the prevalence of anaemia, clinical features, hospitalisation outcomes, and associated factors among paediatric patients with severe malaria-related anaemia admitted to a tertiary hospital in northwestern Nigeria.

**Design:**

This was a retrospective study of children with confirmed severe malaria-related anaemia admitted between 2019 and 2022.

**Participants:**

Paediatric patients aged three months to 14 years with confirmed severe malaria-related anaemia

**Main outcomes measures:**

Hospitalization outcomes and associated factors among paediatric study patients.

**Results:**

There were 278 malaria-related anaemia cases with a prevalence of 29.3% (278/948) among malaria cases and 3.4% (278/8,295) among all paediatric admissions. Of 278 patients with malaria-related anaemia, 110 (39.6%) had severe anaemia. The prevalence of severe anaemia was 11.6% (110/948) and 1.3% (110/8,295) from malaria cases and paediatric admissions, respectively. Clinical features were comparable across the levels of anaemia except for the loss of consciousness (p = 0.038). Severe anaemia was more common among under-fives (76/159, 47.8%), *p*=003, and males (*p* = 0.013). The crude mortality rate was 6.5% (18/278) and comparable [6.4%, (7/110)] with severe anaemia (*p* = 0.924). Factors that were associated with hospitalisation deaths included unconsciousness [adjusted odds ratio (AOR) 5.8, 95% confidence interval (CI) 1.800-18.441], hypoxemia AOR [7.3, 95% CI, 1.749- 30.473] and first 24 hours of admission, AOR [18.4, 95% CI 3.430-98.705].

**Conclusion:**

In childhood, severe malaria anaemia remains a greater burden among under five and is associated with high mortality. Unconsciousness and hypoxemia at presentation and the first 24 hours of admission were associated with increased odds of death.

**Funding:**

None declared

## Introduction

Malaria is endemic in Nigeria, with almost all the population at risk.^1^ Due to the large burden of the disease, Nigeria and ten other countries were targeted for pragmatic approaches for the control and elimination of malaria by the ‘roll back malaria’ and World Health Organization (WHO) and termed ‘high burden, high impact.’^2^ Despite the global and country efforts, Nigeria still ranked number one globally, with a total estimate of 68 million malaria cases and 194,000 deaths in 2021.^3^–^5^ In addition, a large part (80%) of the burden of malaria occurred in children, where it accounts for as high as 20% of deaths among under-fives.^6^ Children are not only vulnerable to malaria infection but also at risk of multiple organ system dysfunction and an increased likelihood of death.^3^,^7^

Among the common clinical manifestations of paediatric severe malaria, especially in holoendemic countries like Nigeria, are the varying levels of anaemia.^8^,^9^ Anaemia from malaria is attributable to some pathogenetic mechanisms, which include rupture of parasitised red cells, immune-mediated breakdown of red cells, dyserythropoietic, splenic destruction of deformed and rigid parasitised red cells, and a depressed bone marrow response despite high-level hypoxia-induced erythropoietin levels.^8^

Although varying levels of malaria-associated anaemia occur in holoendemic environments, mortality is high when the haemoglobin level falls below 3 g/dl, with possible long-term cognitive impairments affecting survival.^9^

The reported prevalence of anaemia in childhood malaria in Nigeria varies from 3.2% to 45%, illustrating regional variations within the country.^9^–^11^ However, worthy of note is the fact that most studies in Nigeria report paediatric malaria-related anaemia along with other features of severe malaria and lack in-depth descriptions of severe malaria-related anaemia.^10^–^14^ In addition, few studies documenting the burden of severe malaria-related anaemia have failed to analyse factors related to hospitalisation outcomes comprehensively.^9^,^11^ Exploring the factors that influence the outcomes of paediatric malaria-related anaemia will provide an opportunity for targeted intervention and ultimately improve outcomes in this vulnerable group. Thus, we hypothesised that anaemia would be high among paediatric admissions with severe malaria in a tertiary health facility in northern Nigeria, and there would be factors associated with poor hospitalisation outcomes. Therefore, we aimed to determine the prevalence of anaemia and describe the clinical features, hospitalisation outcomes (defined as deaths or discharge), and associated factors among paediatric severe malaria admissions at a tertiary health facility in northwestern Nigeria.

## Methods

### Study design and settings

This was a retrospective descriptive study of paediatric malaria-related anaemia managed at a tertiary health facility in northwestern Nigeria from January 1, 2019, to December 31, 2022. The hospital is the only tertiary health facility in the centre of the capital city of Katsina State, one of the seven northern, western Nigeria states with a high burden of seasonal malaria, concomitant malnutrition and associated clinical conditions such as anaemia-related disorders.^15^-^16^ The hospital is a referral centre for sick children from primary and secondary health facilities and serves an estimated population of 8.2 million people.^16^ In addition, the hospital also receives referrals from parts of the adjacent neighbouring states of Kano, Kaduna, Zamfara, and parts of the Niger Republic. The hospital has an emergency paediatric complex with a 28-bed capacity that includes a two-bed high-dependent unit for critically ill children who require close monitoring. The children's emergency was headed by a Consultant Paediatrician and supported by residents and nurses. The people of Katsina mainly practice farming and animal husbandry, with the human development index (0.45) below the national average of 0.54.^16^ The state also has a high burden of malnutrition among the children, with 59% of children under five years of age stunted.^16^

### Sample size estimations

The minimum sample size estimate was based on the 19.1% prevalence of anaemia reported from a previous study, which had a power of 95% and a 5% level of precision.^15^

Using an online sample size calculator (http://www.raosoft.com/samplesize.html), we obtained a minimum sample size of 235. However, all cases of malarial anaemia (278) with complete data within the study period were included.

### Inclusion criteria

This study included children aged between three months and 14 years admitted with a confirmed diagnosis of malaria, either through rapid diagnostic tests or microscopy, and features of severe malaria-related anaemia at admission. We excluded children with other features of severe malaria without anaemia, those with missing haemoglobin or haematocrit data records in their case record file (n = 14), those with sickle cell disease, chronic kidney disease, and those with chronic diseases such as tuberculosis.

### Definition of anaemia

Anaemia in malaria was defined as haemoglobin less than or equal to 11 g/dL and further classified as mild (9–11 g/dL), moderate (6–8 g/dL), and severe (less than or equal to 5 g/dL).^7^

### Patients' management

The management of the children followed the unit protocol, which included a packed cell volume check and complete blood counts as part of routine investigations at the presentation. Each child also received three or more doses of intravenous artesunate (at least the first dose was provided free of charge), along with other appropriate supportive care, including supplemental oxygen in those with hypoxemia and oral artemether-lumefantrine when the children could take it orally after the completion of three doses of intravenous artesunate. Those with a packed red cell volume of 15% and below received a packed cell transfusion at 15 ml/kg, while those with packed red cells between 16 and 22% with evidence of decompensation also received a packed red cell blood transfusion. The children were discharged upon improvement in their clinical conditions.

### Study outcomes

The primary outcomes of this study were hospitalisation outcomes (death or discharge) and associated factors in Children with severe malaria-related anaemia. Other secondary outcomes included a description of the clinical features, levels of anaemia in the study children, and blood transfusion.

### Data collection

The data extracted from the electronic health records included age, sex, admission date, clinical features, haemoglobin level and packed cell volume from the complete blood count, date of outcomes, hospitalisation outcomes (discharged or death), blood transfusion, and time received.

### Ethical consideration and approval

This study was approved by the Federal Medical Center Katsina Health Research Committee (FMCNHREC.REG.N003/0830441) and conducted in accordance with the Declaration of Helsinki. We extracted the data anonymously and kept it confidential using a secure computer.

### Statistical analysis

Data were extracted into an Excel spreadsheet from the hospital's electronic health records by one of the co-authors and verified by another co-author to ensure the accuracy of the information. We subsequently exported and analysed the data using SPSS version 25. Age was not normally distributed and was summarised as the median with an interquartile range (IQR). We summarised the sex distribution and clinical features using frequency tables and compared them using Chi-square and Fischer exact tests, as appropriate. The WHO Anthro® software analysed the weight for age (Z scores) for children ≤ ten years. We evaluated associations between clinical features and outcomes using bivariate analysis (chi-square/odds ratio). Variables significant in bivariate analysis, along with age and sex (independent variables), were entered into binary logistic regression to identify factors associated with hospitalisation deaths (dependent variable). We set the p-value at 0.05 for all levels of statistical significance and reported the odds ratio and adjusted odds ratio with a 95% confidence interval.

## Results

### Sociodemographic characteristics of the study children

A total of two hundred and seventy-eight children were enrolled, with a median age (interquartile range) of 4.0 (2.0–7.0) years. Most children were under five, 149 (57.2%) and males (163; 58.6%). Age was comparable between males and females (Table 1).

**Table 1:** Age and sex distribution of the study children

Variable Age (years)	Total n (%)	Male n (%)	Female n (%)	p-value
Median (IQR)	4.0(2.0-7.0)	4.0(2.0-7.0)	4.0(2.0-6.0)	0.706
< 1	17 (6.1)	11(6.7)	6 (5.2)	0.432
1-<5	142 (51.1)	78 (47.9)	64 (55.7)	
≥ 5	119 (42.8)	74 (45.4)	45 (39.2)	
Total	278 (100)	163 (100)	115(100)	

### Prevalence of anaemia in the study children

During the study period, there were 278 severe malaria cases out of 948 malaria cases and 8,295 paediatric admissions, with a prevalence of 29.3% (278/948) among malaria cases and 3.4% (278/8,295) among all paediatric admissions.

Of the 278 paediatric patients with severe malaria-related anaemia, 110 (39.6%) had severe anaemia, 154 (55.4 %) had moderate anaemia, and 14 (5.0%) had mild anaemia. In overall cases of malaria admission and paediatric admission, the prevalence of severe anaemia was 11.6% (110/948) and 1.3% (110/8,295), respectively.

### Clinical features among the study children

Fever was the most common clinical features among the studied children (257/278; 92.4%), followed by tachycardia (149/278; 53.6%) and tachypnoea (127/278; 45.7%) (Table 2). In addition, most clinical features were comparable across the three levels of anaemia, except for loss of consciousness (*p* = 0.038).

**Table 2:** Clinical features of the study participants

Variables	n=278 (%)	Anaemia			p-value*
	
		Mild n=14	Moderate n=154	Severe n=110	
Age (years)					
**Under-five**	159 (57.2)	5	78	76	0.003
**Five and above**	119 (42.8)	9	76	34	
Sex					
**Male**	163 (58.6)	6	102	55	0.013
**Female**	115 (41.4)	8	52	55	
Fever	257 (92.4)	13	143	101	0.925
Passage of loose stool	19 (6.8)	2	10	7	0.420
Vomiting	43 (15.5)	2	27	14	0.560
Passage of color urine	94 (33.8)	6	48	40	0.507
Loss of consciousness	56 (20.1)	5	36	15	0.038
Multiple convulsions	90 (32.4)	5	53	32	0.622
Oxygen saturation (%)					
**Hypoxemia (≤ 92)**	25 (9.0)	0	17	8	0.367
Respiratory rate					
**Normal**	151 (54.3)	10	88	53	0.155
**Tachypnoea**	127 (45.7)	4	66	57	
Heart/Pulse rate					
**Normal**	129 (46.4)	7	70	52	0.899
**Tachycardia**	149 (53.6)	7	84	58	
Weight for age** (255)					
**<-2 z score**	132 (51.8)	5	69	58	0.564
**-2 to +2 z score**	118 (46.3)	5	71	42	
**> +2 z score**	5 (2.0)	0	2	3	
Duration of illness	3.0	3.0(2.0-3.0)	3.0 (2.0-4.0)	3.0(2.0-5.0)	0.753^K^
Median (IQR)	(2.0-4.0)				
Length of hospitalization					
**≤ 24 hours**	61 (21.9)	2	40	19	0.413
**25 to 72 hours**	111 (39.9)	5	57	49	
**≥ 73 hours**	106 (38.2)	7	57	42	

** Data for children up to 10 years. IQR-Interquartile range

* Fisher exact test; K-Kruskal-Wallis's test

There were significant differences in the three levels of anaemia across age groups, with children under age five having more severe anaemia (76/159; 47.8%) than those aged five and above (34/119; 28.6%). Similarly, there was a significant difference in anaemia levels between the sexes (Table 2). About half (137; 49.2%) of the children were underweight (based on the weight-for-age z-score); however, the weight-for-age z-scores were comparable across the three levels of anaemia.

The median (interquartile range) duration of illness before presentation was 3.0 (2.0–4.0) days and was comparable across the three levels of anaemia. Hospitalisation lasted more than 24 hours for most of the children (172; 61.9%) (Table 2).

### Blood transfusion

**Figure 1:**
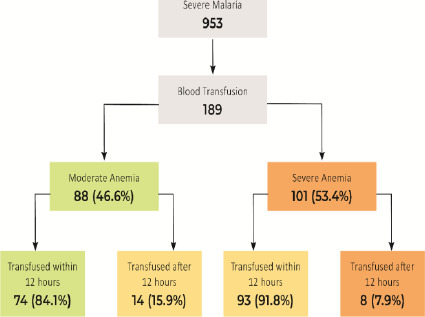
Blood transfusion among the paediatric severe malaria in the study

Of 278 children with anaemia, 68.0% (189) received blood transfusions. Of the 189 patients, 101 (53.4%) had severe anaemia at presentation (Figure 1). Of the 189 patients who received blood transfusions, 167 (88.4%) received transfusions within 12 hours of admission, while the remaining 22 (11.6%) were transfused after 12 hours of admission. Based on the level of anaemia, out of 110 severe anaemia cases, only 101 (91.8%) received a blood transfusion. Of the 101 patients with severe anaemia, 93 (92.1%) received transfusions within 12 hours (Figure 1). Of the 154 children with moderate anaemia, 88 (57.1%) received blood transfusions. Among the 88 patients who received blood transfusions, 74 (84.1%) received blood transfusions within 12 hours of admission (Figure 1).

### Outcomes of hospitalisation

Eighteen of the 278 children died, with a case fatality rate of 6.5%. The mortality rate in severe anaemia (6.4%) was comparable to that in non-severe anaemia (Table 3). The mortality rate among under-fives was 6.9% vs. 5.9% in those aged five and above, p = 0.920. Although mortality was higher in females (9.6%), it was not significantly different from that of their male counterparts (4.3%) (p = 0.088) (Table 3).

**Table 3:** Outcomes of hospitalizations based on age, sex distribution, and anaemia

Variables	Total	Death	Mortality rate (%)	p-value
Age [Years]				
**Under-five**	159	11	6.9	0.809
**Five and above**	119	7	5.9	
Sex				
**Male**	163	7	4.3	0.088
**Female**	115	11	9.6	
Anaemia				
**Severe**	110	7	6.4	1.000
**Non-Severe**	168	11	6.5	

Age and sex were unrelated to hospitalisation outcomes (Table 4). Among the clinical features, after adjusting for confounders, factors that were associated with hospitalisation deaths were unconsciousness at presentation [adjusted odds ratio (AOR) 5.8, 95% confidence interval (CI) 1.800–12.18.441) and hypoxemia (AOR 7.3, 95% CI (1.740–30.473)).

**Table 4:** Factors that are associated with poor hospitalisation outcome (death)

Variables	Cat.	n=18	OR	95% CI	AOR	95% CI	p-value
**Age [Years]**	≥ 5	7	1				
	< 5	11	1.189	0.447, 3.165	1.142	0.375, 3.476	0.815
**Sex**	Female	11	1				
	Male	7	0.424	0.159, 1.130	0.401	0.133, 1.208	0.104
**Fever**	Yes	16	0.631	0.135, 2.949			
**Loose stool**	Yes	2	1.787	0.379, 8.419			
**Vomiting**	Yes	3	1.100	0.304, 3.974			
**Dark urine**	Yes	8	1.619	0.617, 4.248			
**LOC [Yes]**	Yes	8	3.533	1.325, 9.424	5.761	1.800, 18.441	0.003
**Convulsions**	Yes	7	1.357	0.508, 3.626			
**Hypoxemia**	≤ 92	5	4.615	1.494, 14.256	7.281	1.740, 30.473	0.007
**Resp. rate**	Normal	6	1				
	Tachyp.	12	2.522	0.918, 6.924			
**HR/PR**	Normal	6	1				
	Tachyc.	12	1.796	0.645, 4.929			
Weight Z score* (15)	-2 to +2	9	1				
	<-2	6	0.577	0.199, 1.673			
	> +2	0	0.000	0.000			
**Severe anaemia**	No	11	1				
	Yes	7	0.970	0.364. 2.584			
**Transf.**	Yes	15	2.471	0.697, 8.767			
Time of transf. (hrs)*	>12	1	1				
**(15)**	0-12	14	1.922	0.240, 15.372			
**Severity index**	Anaemia	6	1				
	Anaemia +1 feat.	9	2.188	0.654, 7.321			
	Anaemia +2 feat.	3	2.944	0.756, 11.475			
**Sympt. duration (hrs**	≤ 24	4	1				
	25-72	4	0.479	0.116, 1.984			
	> 72	10	1.696	0.507, 5.674			
**LOH (hrs)**	> 72	2	1				
	≤ 24	11	11.440	2.443, 53.573	18.399	3.430, 98.705	0.001
	25-72	5	2.543	0.465, 12.926	3.434	0.618, 19.098	0.159

Cat-category; CI-Confidence interval; OR-odds ratio; AOR-adjusted odds ratio; LOC-loss of consciousness; resp.-respiratory; HR/PR-heart rate/pulse rate; tachyp-tachypnoea; tachyc-tachycardia

* for children ten years and below; transf-transfusion; hrs-hours; sympt-symptoms; LOH-Length of hospitalization. Feat-feature of severe malaria

In addition, the first 24 hours of admission were associated with higher odds of death, with an AOR of 18.4 (95% CI of 3.430 to 98.705), as shown in Table 4. Further sub-analysis showed that of the 18 deaths recorded in this study, 11 occurred within the first 24 hours. Based on the severity of anaemia, three had severe anaemia, while eight had moderate anaemia. The levels of anaemia (moderate vs. severe) were not related to outcomes in the first 24 hours (chi-square, p = 0.737).

## Discussion

Paediatric severe malaria-related anaemia remains a common clinical manifestation in a holoendemic country like Nigeria.^17^ In this study, the prevalence of severe anaemia in children with severe malaria and overall paediatric admissions were 11.6% and 1.3%, respectively. These findings were low compared with a study in Ilorin (northcentral Nigeria), where severe anaemia occurred in 46% of severe malaria and 9.8% of all admissions.^9^ However, our findings were higher than 8.3% (for severe anaemia) in Ibadan (southwestern Nigeria)^11^, but far higher than 3.2% (severe anaemia in malaria) and 1.0% (of total admissions) in Sokoto (northwestern Nigeria).^10^

The values obtained for severe anaemia in this study were less than 22.6 to 28% among children with severe malaria-related anaemia in Ghana.^18^, 24% of children in Cameroun^19^, and 18% among those under five in Kenya.^20^ Our findings confirmed variations in severe anaemia in malaria infection among African children, even though they fell within the observed levels reported in Nigeria and some other African countries. This probably reflects the varying definitions and criteria for severe anaemia, high and variable underlying malnutrition, and related factors. Thus, with a possible superimposed malaria infection, children may have varying degrees of acute severe anaemia.^8^

This finding suggests the need to continue to check for anaemia in children with malaria presenting at various health facilities in Nigeria.

This study also showed an overall mortality of 6.5% and 6.4% among patients with severe anaemia and was comparable across age groups and sexes.

The mortality from severe malaria-related anaemia in this study was lower than that in a Nigerian study (13.2%) in northwestern Nigeria.^10^ and slightly lower to 7.5-8.8% in Kenya.^20^,^21^ It is worth noting that few in-depth studies on the outcomes of severe malaria-related anaemia in Nigeria exist, which limits our further comparison. The lower death rate compared to the study in northwestern Nigeria may also be due to some reasons. First, our sample size for severe anaemia (n = 110) was larger than that of the Nigerian study (n = 38). Second, rapid blood transfusion support services are available; almost 90% of patients with indications for transfusion received blood within 12 hours of admission. Third, as part of the unit protocol, the first doses of artesunate were offered free, which ensured the administration of intravenous artesunate within an hour of presentation, which has been documented to reduce mortality.^3^

The factors related to hospitalisation deaths were unconsciousness and hypoxemia at presentation. In Sokoto, northwestern Nigeria, only cerebral malaria was identified as being associated with hospitalisation death, although hypoxemia and unconsciousness were not evaluated among the independent variables.^10^ The presence of unconsciousness and hypoxemia are some of the clinical findings that highly suggest decompensations during severe anaemia with a high risk of death.^22^ These findings suggest the need for focused attention with appropriate support care, including oxygen therapy among children with hypoxemia and unconsciousness at presentation, to improve outcomes in severe malaria-related anaemia. We also observed higher odds of death in the first 24 hours of admission among the study children. In Kenya, researchers also reported a similar observation of higher hospitalisation mortality within the first 24 hours.^20^ This finding is not unexpected because it is the most critical part of admissions, with most children presenting with many features of severe malaria that may require other supportive care besides blood transfusion.^8^ This observation calls for closer monitoring of children with severe malaria-related anaemia on the first day of admission in addition to providing blood transfusion.

We also observed a comparable clinical feature across the various levels of anaemia except the loss of consciousness, while severe anaemia was common in under-fives and females. The clinical features and observation of anaemia in younger age groups are consistent with the findings in the literature.^10^,^19^ It is worth noting that this age group is vulnerable to severe malaria due to low immunity, the physiological nadir of red cells and blood volume, and a high burden of malnutrition-related anaemia, all of which contribute to a rapid fall in the red cell volume with acute *Plasmodium falciparum* infection.^8^ This finding calls for a sustained target intervention approach for malaria infection prevention and treatment among under-fives, such as using mosquito nets in this age group and prompt treatment of uncomplicated malaria to avoid progression to severe malaria.

### Study limitations

Although this study had a relatively large sample size (n = 278), it has some limitations. Children with severe malaria-related anaemia were not followed up because of limited resources. Follow-up of these children would have allowed for assessing the levels of haemoglobin post-discharge, especially among those with mild to moderate anaemia. We also did not investigate the possible role of metabolic acidosis, which has been known to be associated with poor outcomes in children with severe malaria-related anaemia. In addition, this is single tertiary health facility data from northern Nigeria and may not be a true reflection of the country. In addition, fourteen (14) children did not have haemoglobin or hematocrits from their records and were excluded (given the retrieval rate of 95.2% of the records of the study children).

## Conclusion

In childhood, severe malaria and anaemia remain high, with a greater burden among under-fives, and are associated with high mortality. The presence of unconsciousness and hypoxemia at presentation and the first 24 hours of admission were associated with increased odds of death among children with severe malaria-related anaemia. In addition to prompt blood transfusion where indicated, attention should be paid to children with severe malaria-related anaemia in the first 24 hours of admission.
